# Machine Learning-Guided Prediction of Antigen-Reactive In Silico Clonotypes Based on Changes in Clonal Abundance through Bio-Panning

**DOI:** 10.3390/biom10030421

**Published:** 2020-03-08

**Authors:** Duck Kyun Yoo, Seung Ryul Lee, Yushin Jung, Haejun Han, Hwa Kyoung Lee, Jerome Han, Soohyun Kim, Jisu Chae, Taehoon Ryu, Junho Chung

**Affiliations:** 1 Department of Biochemistry and Molecular Biology, Seoul National University College of Medicine, Seoul 03080, Korea; dk93js@gmail.com (D.K.Y.); seungryul93@gmail.com (S.R.L.); intia6@gmail.com (H.K.L.); wsukh23@gmail.com (J.H.); kimchii481@gmail.com (S.K.); jschaelab@gmail.com (J.C.); 2 Department of Biomedical Science, Seoul National University College of Medicine, Seoul 03080, Korea; 3 Cancer Research Institute, Seoul National University College of Medicine, Seoul 03080, Korea; 4 Celemics, Inc., 131 Gasandigital 1-ro, Geumcheon-gu, Seoul 08506, Korea; yushinjung@celemics.com (Y.J.); hhjunny@gmail.com (H.H.)

**Keywords:** c-Met, next-generation sequencing, phage display, machine learning, random forest, antibody discovery

## Abstract

c-Met is a promising target in cancer therapy for its intrinsic oncogenic properties. However, there are currently no c-Met-specific inhibitors available in the clinic. Antibodies blocking the interaction with its only known ligand, hepatocyte growth factor, and/or inducing receptor internalization have been clinically tested. To explore other therapeutic antibody mechanisms like Fc-mediated effector function, bispecific T cell engagement, and chimeric antigen T cell receptors, a diverse panel of antibodies is essential. We prepared a chicken immune scFv library, performed four rounds of bio-panning, obtained 641 clones using a high-throughput clonal retrieval system (TrueRepertoire^TM^, TR), and found 149 antigen-reactive scFv clones. We also prepared phagemid DNA before the start of bio-panning (round 0) and, after each round of bio-panning (round 1–4), performed next-generation sequencing of these five sets of phagemid DNA, and identified 860,207 HCDR3 clonotypes and 443,292 LCDR3 clonotypes along with their clonal abundance data. We then established a TR data set consisting of antigen reactivity for scFv clones found in TR analysis and the clonal abundance of their HCDR3 and LCDR3 clonotypes in five sets of phagemid DNA. Using the TR data set, a random forest machine learning algorithm was trained to predict the binding properties of in silico HCDR3 and LCDR3 clonotypes. Subsequently, we synthesized 40 HCDR3 and 40 LCDR3 clonotypes predicted to be antigen reactive (AR) and constructed a phage-displayed scFv library called the AR library. In parallel, we also prepared an antigen non-reactive (NR) library using 10 HCDR3 and 10 LCDR3 clonotypes predicted to be NR. After a single round of bio-panning, we screened 96 randomly-selected phage clones from the AR library and found out 14 AR scFv clones consisting of 5 HCDR3 and 11 LCDR3 AR clonotypes. We also screened 96 randomly-selected phage clones from the NR library, but did not identify any AR clones. In summary, machine learning algorithms can provide a method for identifying AR antibodies, which allows for the characterization of diverse antibody libraries inaccessible by traditional methods.

## 1. Introduction

The mesenchymal-epithelial transition factor (c-Met) and its ligand hepatocyte growth factor (HGF) are well-known oncogenic drivers of tumorigenesis [1]. Numerous clinical observations have demonstrated that c-Met overexpression or gene alterations play a key role in both oncogenesis and the development of drug resistance across multiple cancer types [2,3,4,5]. Furthermore, recent research suggests that the HGF-c-Met axis limits the efficacy of cancer immunotherapy by modulating immune cell function and the expression of programmed cell death ligand 1 (PD-L1) [6,7,8,9]. Despite efforts to inhibit the HGF-c-Met axis including antibodies against c-Met or HGF, c-Met tyrosine kinase inhibitors, and more, no therapeutic agent specific to the HGF-c-Met axis is clinically available. Currently, two anti-HGF antibodies, including YYB-101 previously discovered by our group, are under clinical trials (NCT02499224) [10]. However, no antibodies are under development against c-Met after the failure of onartuzumab in clinical trials [11].

Based on rapid advances in next-generation sequencing (NGS) technology, various methodologies for analyzing NGS data have been developed to decode the antibody repertoire from diverse sources such as the natural B cell receptor of animals and humans as well as recombinant antibody libraries that can be synthetically designed and constructed [12,13,14]. Furthermore, combining surface display technology and NGS analysis offers synergistic advantages in identifying antigen-reactive clones in silico over the laborious in vitro screening process, which is frequently overwhelmed by dominant antibody clones [15]. Traditional bio-panning methodologies are biased towards the excessive enrichment of dominant clones with significant suppression of antibody diversity. Consequently, this approach could lead to the omission of potential antigen-reactive (AR) clones with low clonal abundance or their diminishment by unintended selective pressure. 

Previously, our group analyzed the enrichment patterns of bio-panned clones by employing NGS technology to predict the antigen binding properties of antibody clones inside different clusters [16]. First, we tracked the clonal abundance of heavy chain complementarity region 3 (HCDR3) through multiple rounds of bio-panning with NGS analysis, and then applied clustering analysis to group HCDR3 clonotypes based on the enrichment pattern. As a result, different clusters (enriched, impoverished, and fluctuated) were generated with the enriched pattern cluster containing a higher frequency of AR scFv (single-chain variable fragment) clones than other clusters. However, due to limitations in retrieving the physical DNA of encoded scFv from a large, diverse number of clones, we were unable to sufficiently observe the binding properties of in silico scFv clones. Recently, we developed a laser and microchip-based high-throughput clonal retrieval system (TrueRepertoire^TM^, TR) for scFv DNA from the library [17], which allows a much higher number of scFv clones to be obtained and tested for antigen reactivity. 

In this study, we established a phage-displayed chicken scFv library after immunization with recombinant c-Met. Four rounds of bio-panning were performed on antigen-conjugated magnetic beads. Through bio-panning, five sets of phagemid DNA (rounds 0–4) were obtained and subjected to NGS analysis using both HiSeq and MiSeq platforms. After the final round of bio-panning, scFv-displayed phage clones were obtained in a high-throughput manner using TR technology, and individual clone reactivity was evaluated by phage enzyme-linked immunosorbent assay (ELISA). From NGS data obtained using the HiSeq platform, HCDR3, and light chain complementarity region 3 (LCDR3) clonotypes were extracted and evaluated for their clonal abundance in phagemid DNA sets from round 0 (before biopanning) to round 4. We then established a data set (TR data set) containing the antigen reactivity of scFv clones retrieved through TR technology and the clonal abundance of their HCDR3 and LCDR3 clonotypes in five sets of phagemid DNA. Using this TR data set, we trained our random forest (RF) machine learning algorithm to predict the binding properties of in silico HCDR3 and LCDR3 clonotypes [18,19]. 

**Figure 1:**
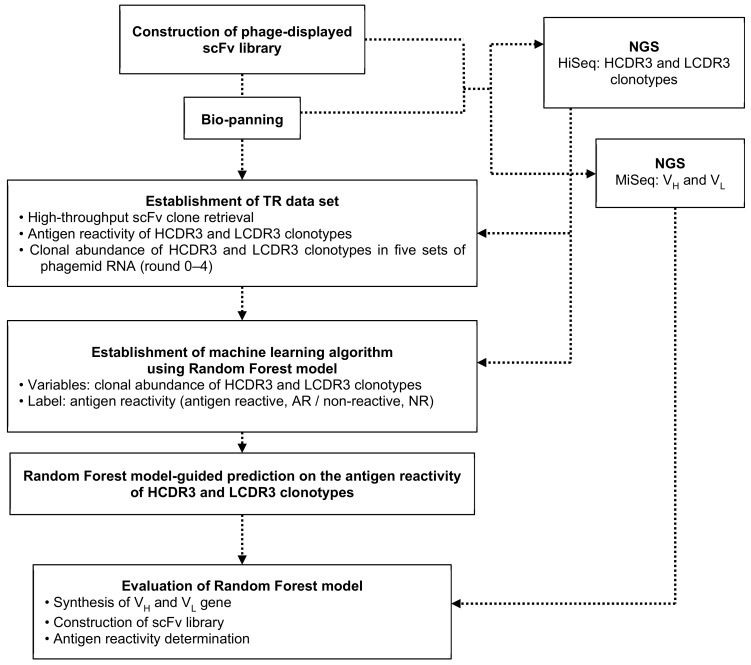
Workflow of the machine learning-guided selection of antigen-reactive HCDR3 and LCDR3 clonotypes with confirmation of their reactivity.

To test the accuracy of our RF model (Figure 1), we extracted V_H_ and V_L_ sequences from MiSeq NGS data, which encompass both RF model-determined AR and antigen non-reactive (NR) HCDR3 or LCDR3 clonotypes and chemically synthesized them. Using these V_H_ and V_L_ genes, we established two phage-displayed scFv libraries. The AR library was prepared using V_H_ and V_L_ genes encompassing AR HCDR3 and LCDR3 clonotypes, and the NR library was constructed using V_H_ and V_L_ genes encompassing NR HCDR3 and LCDR3 clonotypes. After one round of bio-panning on antigen-conjugated magnetic beads, antigen reactivity of phage clones was tested by phage ELISA. From the AR library, we obtained many scFv clones containing AR HCDR3 and LCDR3 clonotypes, while no AR clones were enriched from the NR library.

## 2. Materials and Methods 

### 2.1. Immunization, Construction of Phage-Displayed scFv Library, and Bio-Panning

White leghorn chickens were immunized and boosted three times with 10 µg of recombinant mouse c-Met-Fc chimera (527-ME; R&D systems, Carlsbad, CA, USA). The experiment was approved by the Ethics Committee of BioPOA, Ltd. (ethical approval code: BP-2019-C03-1). One week after the final boosting, total RNA was isolated from spleen, bone marrow, and bursa of Fabricius using TRIzol Reagent (15596018; Invitrogen), and cDNA was synthesized using SuperScript III first-strand cDNA synthesis kit with oligo dT priming (18418020; Invitrogen, Carlsbad, CA, USA). Using this cDNA, a phage-displayed scFv library was prepared as described previously [20,21]. V_H_ and V_L_ genes were amplified from the cDNA using specific primer sets utilized for the construction of scFv genes. Then, scFv genes were ligated into the pComb3XSS phage display vector, which was transfected into *E. coli* K12 ER2738 cells. Phage-displayed scFv libraries were rescued from transfected cells after infection with VCSM13 helper phage and overnight culture, and then subjected to four rounds of bio-panning using recombinant mouse c-Met (50622-M08H, Sino Biological, Beijing, China)-conjugated magnetic beads (Dynabeads 14302D; Invitrogen). Antigen-coated magnetic beads were washed with 0.05% tween in phosphate-buffered saline (PBS) once for the first round, three times for the second and third rounds, and five times for the fourth round. After each round of bio-panning, phagemid DNA was prepared from bacterial cell pellets using a Qiaprep Spin Miniprep Kit (27104, Qiagen, Hilden, Germany).

### 2.2. Next-Generation Sequencing (NGS) 

From five sets of phagemid DNA, short V_H_ and V_L_ gene fragments encoding the 3′ part of FR3 and CDR3, and the 5′ part of FR4, were amplified using primers designed to hybridize to FR3 and FR4 of the chicken V_H_ gene (LFR3: 5′-CCCTTCACGATTCTCCGGTGCC-3′; LFR4: 5′-CTGACCTAGGACGGT CAGGG-3′; HFR3: 5′-GGCTGCAGCTGAACAACCTCAGGGCTG-3′; HFR4: 5′-GGAGGAGACGA TGACTTCGGTCCCGTGG-3′). Other gene fragments encoding the whole V_H_ and V_L_ genes were also amplified using specific primers previously described [16]. Prior to NGS analysis, all amplicon libraries were submitted for a quality control procedure on TapeStation 2200 (Agilent Technologies, Santa Clara, CA, USA). Libraries having a single peak of correct fragment length were subjected to NGS analysis using the HiSeq 2500 and MiSeq platforms (Illumina, Inc.) for short and whole V_H_ and V_L_ gene fragments, respectively. We uploaded the sequence data to NCBI (SRA accession number: PRJNA607865). 

To ensure the quality of NGS data, the following pre-processing steps were performed. First, all pair-end reads were merged with PEAR using the developer’s default parameters [22]. Second, we filtered out any reads that were compatible with the following description: (1) reads not meeting our minimum quality Phred score, (2) reads not having the primer sequence used in the phage-displayed scFv library construction process, (3) out-of-frame reads, and (4) reads without any identifiable CDR3. The reads were then collated based on their CDR3 sequences and any CDR3 clonotype with read count of less than 2 was discarded.

### 2.3. High-Throughput Clone Retrieval and Phage ELISA

The phagemid library from the final bio-panning round was transfected into *E. coli* K12 ER2738 cells, and then subjected to our high-throughput clonal retrieval procedure using TrueRepertoire (TR) technology, as described previously [17]. 

The retrieved phage clones were subjected to phage ELISA, as described previously with adequate modifications [21]. Phage clones were rescued overnight from the plate and culture supernatants containing phage that were diluted with equal volumes of 6% bovine serum albumin (BSA) solution in PBS. Phage solutions were then added to microtiter wells (3690, Corning life sciences, Corning, New York, NY, USA) coated with recombinant mouse c-Met or mouse anti-HA antibody (H3663, Merck, Darmstadt, Germany) and blocked with BSA. Microtiter plates were incubated for 2 h at 37 °C and washed three times with 0.05% Tween in PBS, which is followed by 3% BSA in PBS containing horseradish peroxidase (HRP)-conjugated anti-M13 antibody (11973-MM05, Sino Biological) in addition to each well. After incubation and washing as described above, HRP substrate solution 2,2’-azino-bis(3-ethylbenzothiazoline-6-sulfonic acid) (ABTS) (002024, Thermo Fisher Scientific, Waltham, MA, USA) was added to each well. The plate was incubated 15 min and the absorbance values of each well were measured by a SkanIt microplate reader (Thermo Fisher Scientific) with a fast measurement protocol at a wavelength of 405 nm. 

For each clone, the ratio (Relative Absorbance A) of the average absorbance of a recombinant mouse c-Met-coated well vs. an anti-HA antibody-coated well was calculated. The absorbance of an anti-HA-coated well was used to accommodate variations in the amount of phage in each phage clone. We also determined the ratio (Relative Absorbance B) of the average absorbance of a BSA-blocked well to an anti-HA antibody-coated well. When Relative Absorbance A exceeded +3 standard deviation of Relative Absorbance B, we designated the phage clone as antigen-reactive. 

### 2.4. Establishment of the Random forest (RF) Models

Random forest (RF), regularized discriminant analysis (RDA), linear discriminant analysis (LDA), support vector machine (SVM), naïve bayes (NB), and AdaBoost (ADA) classification trees were selected for comparison. Our input data for the training of binder prediction models were created using a TR data set consisting of antigen reactivity for scFv clones found in TR analysis and the clonal abundance of their HCDR3 and LCDR3 clonotypes in five sets of phagemid DNA. The caret package for R was used to benchmark popular classification algorithms by their accuracy and Cohen’s kappa value. Each algorithm was evaluated across five repetitions of 10-fold cross-validations (50 models in total). This meant that, for each repetition, the training data set was randomly divided into 10 parts and each of the 10 models were cross-validated by one unique part after being trained on the other nine parts of the training data set. No manual tuning was performed during this benchmarking phase [23].

To generate binder prediction models for HCDR3 and LCDR3 clonotypes using a random Forest package, we sampled a proportion of the TR data set without replacement to be used as a training data set for the RF model. The remaining portion of the TR data set served as a validation set to measure the performance of RF models [24]. The following parameters were adjusted to best tune our model’s performance: (1) sampling ratio of training data, (2) number of variables (mtry) to randomly sample at each node of the decision-making tree, and (3) number of trees (ntree) to compromise our RF model. We then iterated through all combinations of parameters. Each combination was used to generate 10 different RF models to minimize any biases arising from the training data set not being representative of the TR data set. The validation set was then used to measure the performance of each RF model to determine optimal parameters for the RF. Using the randomForestExplainer package, the minimum depth of each variable was calculated, which is frequently used as a measure of variable importance to elucidate the decision-making process of the algorithm [25]. The minimum depth of a variable is defined as the distance between the root node and the variable’s first appearance at a node of the decision tree. Thus, the variable with the smallest mean minimum depth could be regarded as the most important variable. To compare the variable importance results of the prediction model with actual experimental data, we tracked the enrichment pattern by measuring the bio-panning titer and clonal diversity change as Shannon’s entropy (SE) [26] by following each round of bio-panning, as described previously [21].

### 2.5. Construction of Antigen-Reactive (AR) and Non-Reactive (NR) Phage-Displayed scFv Library and Phage ELISA

Forty antigen-reactive (AR) and 10 non-reactive (NR) V_H_ and V_L_ genes were chemically synthesized (Twist Bioscience, San Francisco, CA, USA). Forty AR V_H_ and 40 AR V_L_ genes were subjected to linker PCR to generate scFv genes, which were used to create the AR phage-displayed scFv library, as described previously [20]. In a parallel experiment, the NR phage-displayed scFv library was constructed using 10 NR V_H_ and 10 NR V_L_ genes. After a round of bio-panning using recombinant mouse c-Met (50622-M08H; Sino Biological)-conjugated magnetic beads (Dynabeads 14302D; Invitrogen) and washing once with 0.05% tween in PBS, 96 phage clones were randomly rescued from each AR and NR library and subjected to phage ELISA, as described above. After phage ELISA, the nucleotide sequences of scFv clones were determined by Sanger nucleotide sequencing (Macrogen, Seoul, South Korea). 

## 3. Results

### 3.1. Construction of Phage-Displayed scFv Library, Biopanning, Selection of Positive Clones, Next-Generation Sequencing (NGS), And Establishment of TR Data Set

Chickens were immunized with recombinant mouse c-Met-Fc chimera. Spleen, bone marrow, and bursa of Fabricius were harvested from the immunized chickens and total RNA was prepared to generate a phage-displayed scFv library with a complexity of 4.96 × 10^9^. Four rounds of bio-panning were performed using antigen-coated magnetic beads. After the final round of bio-panning, the phage pool was subjected to high-throughput clonal retrieval using TR technology. From the TR analysis, 641 clones with unique V_H_ and V_L_ pairs were identified. These phage clones were rescued and subjected to phage ELISA. Out of 641 phage clones, 149 clones showed reactivity to c-Met with statistical differences from non-reactive clones (data not shown) designated as AR clones. We used the binding reactivity of the 641 clones as a part of the TR data set.

After arranging the phage-displayed scFv library and each round of bio-panning, phagemid DNA (rounds 0–4) was prepared using bacterial pellets obtained after centrifugation of overnight culture supernatant. From these five sets of phagemid DNA, gene fragments encoding HCDR3 and LCDR3 were amplified and subjected to NGS analysis using the HiSeq platform. After NGS data pre-processing, we defined valid clonotypes as unique CDR3 sequences with read counts of two or higher in any set of phagemid DNA, and we were able to retrieve 860,207 HCDR3 clonotypes and 443,292 LCDR3 clonotypes across the entire bio-panning phase (Table 1). Clonal abundance throughout bio-panning stages was determined by counting the number of times that a clonotype appeared in each bio-panning round. The clonal abundance of clonotypes matching to scFv clones found in TR analysis was used as another part of the TR data set. We also amplified V_H_ and V_L_ gene fragments from five sets of phagemid DNA and subjected them to NGS analysis using the MiSeq platform.

### 3.2. Establishing Random Forest (RF) Binding Reactivity Prediction Model

We compared random forest, regularized discriminant analysis, linear discriminant analysis, support vector machine, naïve bayes, and AdaBoost classification trees for their accuracy and kappa score distributions. We found out that the random forest algorithm was best suited for binder predictions of HCDR3 clonotypes with the mean accuracy of 89.69% and mean Cohen’s kappa value of 0.45 (Tables S1–S4 and Figure S1). While regularized discriminant analysis did perform marginally better in the LCDR3 clonotypes, random forest showed more potential for improvement with manual tuning when consulting maximum accuracy and Cohen’s kappa value. With these observations, we decided to adopt random forest models to establish a binding reactivity prediction model. 

Utilizing the TR data set, two separate RF models were trained for HCDR3 and LCDR3 clonotypes. The algorithm was instructed to treat the clonal abundance of clonotypes in the five sets of phagemid DNA (round 0–4) as predictor variables and the binding reactivity as the response variable. Thus, each unique clonotype in our TR data set was individually labelled with that clonotype’s abundance at each of the bio-panning rounds and its binding reactivity. Before the training of each new RF model, the TR data set was divided into a training data set and a validation data set. After training the RF model using the training set, the validation set was presented to the RF model, and RF model accuracy in predicting clonotype binding reactivity was determined. 

To determine the optimum training parameters for our RF model, 7200 RF models were evaluated. Optimizing for sensitivity, the ideal parameters for the HCDR3 RF model were found to be a 75% sampling ratio of the TR data set, mtry of 4, and ntree of 500. The performance metrics of 10 RF models using those parameters were: (1) mean accuracy of 90.48%, (2) mean sensitivity of 44.36%, and (3) mean specificity of 97.61%. Optimizing for accuracy, the ideal parameters for the LCDR3 RF model were found to be a 65% sampling ratio of the TR data set, mtry of 2, and ntree of 500. Once again, the performance metrics of 10 LCDR3 RF models using those parameters were: (1) mean accuracy of 86.47%, (2) sensitivity of 55.98%, and (3) specificity of 94.90% (Table S5). 

### 3.3. Measurement of the Minumum Depth Value of a Predictor Variable 

**Figure 2:**
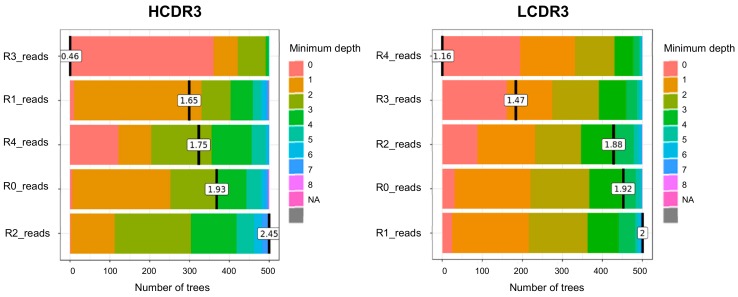
Distribution of the minimum depth of predictor variables (clonal abundance at round 0–4 of bio-panning) from individual decision trees in the RF prediction model for CDR3 clonotypes. Minimum depth value is colored according to its depth and mean value is calculated and displayed at points.

The minimum depth of a predictor variable can be interpreted as a measure of the variable importance. We extracted the minimum depth value of each predictor variable from the 500 decision trees that compromised our RF model. Of note is that, in our HCDR3 RF model, our predictor variable representing CDR3 clonal abundance in round 3 of bio-panning was most likely to appear at the root node of our decision trees appearing in 360 instances out of our 500 decision trees, and, consequently, had the lowest mean minimum depth of 0.46. In our LCDR3 RF model, our predictor variable representing CDR3 clonal abundance in round 4 of bio-panning was most likely to appear at the root node of our decision trees appearing in 195 instances out of 500 decision trees and, consequently, had the lowest minimum depth of 1.16 (Figure 2). In accordance with these observations, the Shannon entropy (SE) representing clonal diversity dropped at round 3 in the case of HCDR3 clonotypes while the SE of LCDR3 significantly dropped at round 4 (Table S6 and Figure S2). 

**Figure 3:**
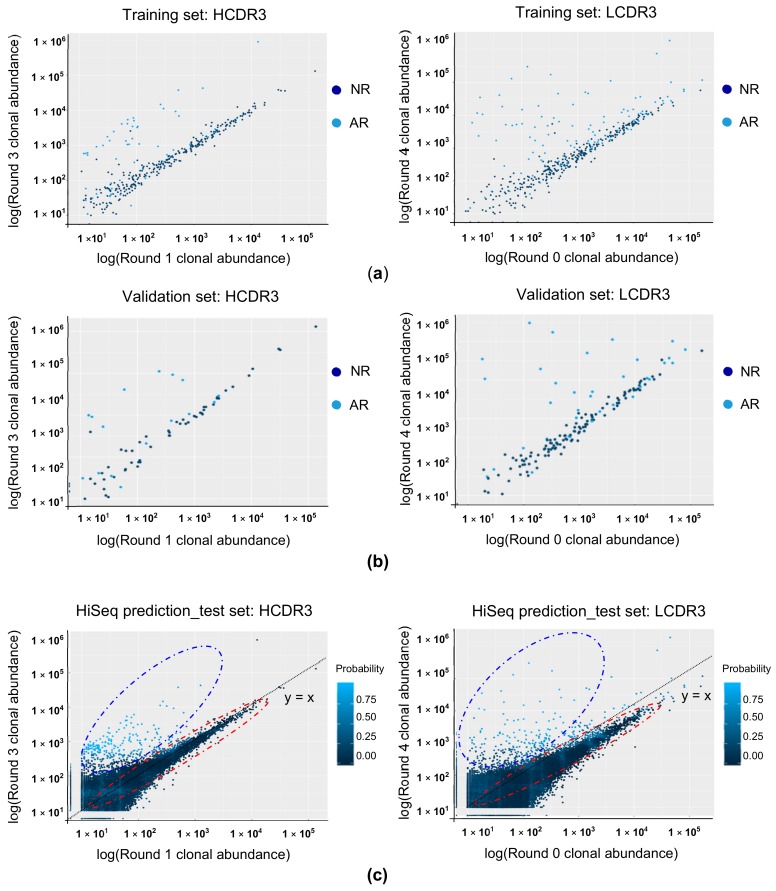
The most influential variable interactions and distributions of CDR3 clonotypes. (**a**) Clonal abundance at the most influential interaction is plotted with binding property label from training data used in the random forest (RF) prediction model. AR, antigen-reactive, NR, antigen non-reactive. (**b**) Clonal abundance at the most influential interaction is plotted with a binding property label from validation data used in the RF prediction model. (**c**) Clonal abundance at the most influential interaction is plotted with confidence value (probability) from HiSeq-identified CDR3 clonotypes. Clonotypes with higher confidence values are distributed near the root variable axis (highlighted with a dashed blue circle) while clonotypes having lower confidence values are distributed below the y = x axis (dotted line) (highlighted with a dashed red circle).

We also observed the interaction of our predictor variables taking place within the decision trees. Variable interactions are regarded as taking a sub-tree of two nodes and considering it as a single node. We can then look at the minimum depth value of that sub-tree to gauge the interaction’s importance in classifying its input. In our HCDR3 RF model, the top four most influential interactions all involved clonal abundance in round 3 of bio-panning as the root node. The most influential interaction took place between round 3 and round 1 with a minimum depth value of 0.84 (Table S7). In our LCDR3 RF model, three of the top four most influential interactions involved clonal abundance in round 4 of bio-panning as the root node. The most influential interaction took place between round 4 and round 0 with a minimum depth value of 1.18 (Table S8). Using the training data set, the clonal abundance of HCDR3 clonotype in round 3 and round 1 and that of LCDR3 clonotype in round 4 and round 0 were plotted in Figure 3a,b, which shows significant correlation.

### 3.4. Predicting the Binding Property of the CDR3 Clonotype Using RF Modeling

Of the 860,207 HCDR3 clonotypes fed into the RF model, 5,780 clonotypes were predicted to be AR. Of the 443,292 LCDR3 clonotypes, 34,703 clonotypes were predicted to be AR. The confidence value of the RF model for each prediction was also obtained. For HCDR3 and LCDR3, 1.70% (98/5,780) and 0.16% (58/34,703) of clonotypes, respectively, were predicted to be AR with a confidence value of more than 0.9. Meanwhile, 0.56% (4,825/854,427) of HCDR3 clonotypes and 41.14% (168,116/408,589) of LCDR3 clonotypes were predicted to be NR with a confidence value over 0.9. When CDR3 clonotypes were visualized with the most important variable interactions together including a confidence value (Figure 3c), clonotypes with higher confidence values were distributed near the axis of the most important variable akin to the distribution of AR clonotypes in the training data set. 

### 3.5. Antigen Reactivity Validation of In Silico CDR3 Clonotypes in Phage ELISA

**Figure 4:**
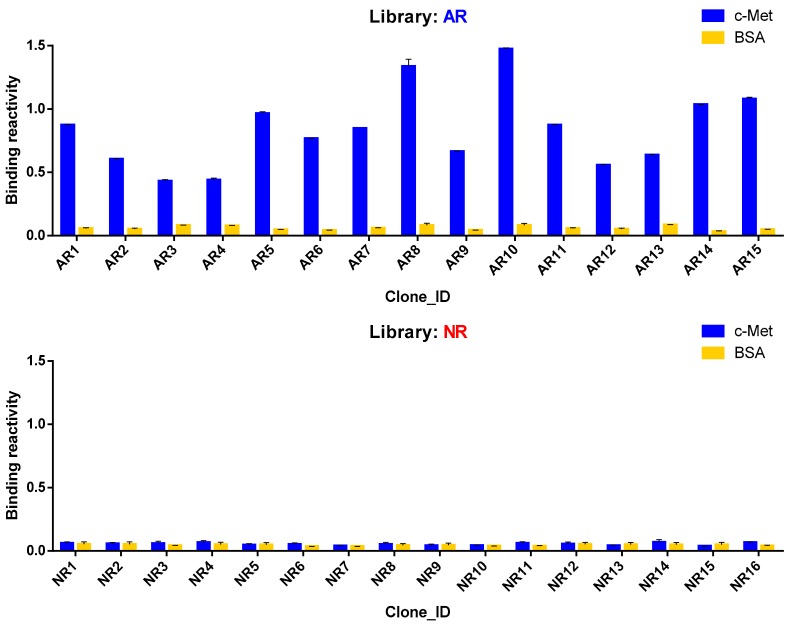
Reactivity of phage-displayed scFv clones in phage ELISA. Binding reactivity of 15 unique clones identified from the AR library and 16 unique clones from the NR library are shown. Wells in microtiter plates were either coated with recombinant mouse c-Met or just blocked with 3% BSA in PBS. Phage clones, HRP-conjugated anti-M13 antibody, and HRP substrate solution were added sequentially with intermittent washing.

We selected 40 HCDR3 AR, 40 LCDR3 AR, 10 HCDR3 NR, and 10 LCDR3 NR clonotypes with the highest confidence values of which whole V_H_ or V_L_ gene sequences were available from the NGS data generated from five sets of phagemid DNA using the MiSeq platform (Tables S9 and S10). After whole V_H_ and V_L_ genes were chemically synthesized, V_H_ and V_L_ genes of AR clonotypes were used to construct the AR phage-displayed scFv library. In a parallel experiment, the NR phage-displayed scFv library was also constructed using the same scheme. After a single round of bio-panning on antigen-coated magnetic beads, 96 phage clones were randomly selected from the output titer plate of the AR library and subjected to phage ELISA. Fifteen phage clones were found to be AR, which turned out to be 14 scFv clones consisting of five HCDR3 and 11 LCDR3 clonotypes by Sanger sequencing (Figure 4, Table 2). AR5 and AR6 phage clones encoded the same scFv sequence. It was noticeable that three LCDR3 clonotypes were paired with two different HCDR3 clonotypes as in AR1 and AR13, AR2, and AR7, and AR4 and AR14 phage clones showing light chain redundancy. In a parallel experiment, no AR clones were identified from 96 phage clones from the NR library. Sixteen clones were randomly selected and Sanger sequencing was performed to find 13 HCDR3 and nine LCDR3 clonotypes. With these results, we concluded that our RF model can be used to select HCDR3 and LCDR3 AR clonotypes with a significant hit ratio.

## 4. Discussion

Despite the promise of targeting the HGF-c-Met signaling pathway for cancer therapy, no specific therapeutic agent has been approved for clinical use. Small molecule inhibitors specific to c-Met are yet to be approved, and only nonspecific tyrosine kinase inhibitors inhibiting c-Met are available (Table 3) [27]. Recombinant protein (truncated HGF, decoy c-Met) was not successful in clinical trials due to several factors, including short half-life and low target affinity limiting the intended efficacy [28]. Several HGF-neutralizing antibodies have been developed with two currently active in clinical trials [29]. However, the inhibitory targeting of c-Met by an antibody has been difficult since the bivalency of antibodies often induces receptor dimerization, which potentially causes cancer cell proliferation and migration. As such, both a monovalent form of antibody blocking its interaction with HGF and a bivalent antibody inducing receptor internalization have been developed and tested in clinical trials unsuccessfully [13,30]. Recently, an anti-EGFR x c-Met bispecific antibody monovalent to each target came under clinical development, which should inhibit the ligand interaction and induce the internalization of both receptors [31,32]. Besides blocking the interaction with ligand and receptor internalization, other mechanisms of actions for therapeutic antibody binding to targets on cancer cells were also reported, which include complement-dependent cell cytotoxicity as observed in rituximab [33], antibody-dependent cell cytotoxicity seen with obinutuzumab [34], and phagocytosis of antibody-opsonized tumor cells [35]. Antibodies are also used to deliver cytotoxic payloads into cancer cells such as with T-DM1 [36], and cross-linking cancer cells to cytotoxic T cells with blinatumomab [37]. Furthermore, antibodies are used as a cancer cell-targeting component in chimeric antigen receptor T cell therapy, as seen with tisagenlecleucel and axicabtagene ciloleucel [38]. Additionally, it is well known that the antibody epitope and binding characteristics critically influence efficacy for all these various modes of action [39,40]. Therefore, it is crucial to develop a significant number of antibodies to a target and characterize their performance. However, antibody selection technologies, including conventional hybridoma and display technologies such as phage, ribosomal, and bacterial, all have their own limitations regarding high-throughput capabilities. 

After George P. Smith and Gregory P. Winter successfully displayed recombinant peptides and antibodies at the pIII protein of the M13 phage [41], this powerful technology has evolved and been actively applied toward therapeutic antibody discovery [42,43]. Currently, over 80 antibodies derived from phage display libraries have entered clinical studies with 10 of these granted marketing authorization [44]. Since Ravn U et al. demonstrated the potential for NGS analysis in the phage-displayed antibody repertoire in 2010, numerous groups have leveraged similar strategies for discovering antibodies reactive to specific antigens [16,45,46,47,48,49,50,51,52,53]. The next hurdle to overcome after the identification of in silico antibody sequences in NGS data was the low-throughput nature of chemically synthesizing all antibody sequences and individually testing their reactivity. Recently, we introduced a method for combining NGS analysis and individual antibody sequence identification with the isolation of their physical DNA, which was named TR technology [17]. To reduce the burden of expressing all of the antibodies, we also devised a way of predicting antigen reactivity toward antigens by clustering antibody clonotypes with their patterns of enrichment or restriction through bio-panning rounds, and then combining TR with clustering and testing reactivity for a significant number of clones.

Using these tools and procedures, we believed that it was possible to train a machine learning algorithm to derive in silico AR clonotypes from a repertoire of NGS sequences. To demonstrate this, we performed an in-depth analysis of our bio-panning library with the guidance of our supervised machine learning algorithm trained with large amounts of data sets generated from a high-throughput clone retrieval platform and independent NGS analysis. The RF model utilized is composed of numerous unique decision trees that work together to classify inputs. Each decision tree in an RF model is generated using a bootstrapped sample of the training data and a randomized subset of variables evaluated for the best split at each node of that decision tree. As a result, each RF model decision tree is uniquely generated and makes the model more robust to overfitting compared to other linear classifiers or decision trees. Compared to the more complicated black boxes of artificial neural networks, RF models frequently show similar levels of predictive performance while remaining observable and transparent. By inspecting the composition of decision trees in the RF model, we can extract important measures of input variables to better understand the decision-making process of the algorithm. Our extraction of variable importance measures helped explain the logical processes of our RF prediction model, which consists of complex, randomized interactions of predictor variables and response variables. From these results, we can infer that AR HCDRs are mostly selected in enrichment rounds, while LCDR3s are significantly enriched with selected HCDR3s after additional selective pressure occurs. We can then infer that enrichment of scFv molecules depends on individual chains in different stages of the bio-panning process (first V_H_ is then significantly biased by V_L_). We believe our prediction model may be enhanced to better predict binding reactivity with multiple (high, mid, low) rather than binary (reactive/non-reactive) classifications. It is highly likely that this model can be applied to other display platforms that use bio-panning as the selection process, such as yeast display library for fluorescence-activated cell sorting screening [54]. Recently, artificial intelligence has been applied to predict the physicochemical properties of antibody sequences [55,56,57,58,59] and/or optimize them [60,61,62]. 

In summary, we report that machine learning algorithm can provide a way to identify AR antibody clones with a significant hit ratio, which will allow us to better characterize diverse antibodies in greater numbers currently unattainable by traditional methods. 

## Figures and Tables

**Table 1:** Number of CDR3 clonotypes obtained from the bio-panning procedure.

Clonotypes	Round 0	Round 1	Round 2	Round 3	Round 4	Total
HCDR3	390,814	395,459	402,854	311,678	308,547	860,207
LCDR3	272,317	253,899	250,630	187,314	117,239	443,292

**Table 2:** Amino acid sequences of AR CDR3 clonotypes identified from AR library.

Clone ID	HCDR3 AA * Sequence	LCDR3 AA * Sequence
AR1	GSGGVDSIDA	GSYDNTYAGI
AR2	SADGYGWDTAGNMDA	GSIDSNYDGI
AR3	TAGTCTTSCNAGAYIDA	GGYDGSSAA
AR4	TTCSGSYGWCADSIDA	GAYDSSYIGI
AR5	SADSCATCATYPSEIDT	GSFDSSYVGM
AR6	SADSCATCATYPSEIDT	GSFDSSYVGM
AR7	SADSCATCATYPSEIDT	GSIDSNYDGI
AR8	SADSCATCATYPSEIDT	GSYDSSYVGL
AR9	SADSCATCATYPSEIDT	GSYDSSYDGV
AR10	SADSCATCATYPSEIDT	GSFDSSYTGI
AR11	SADSCATCATYPSEIDT	GSIDSRYVGI
AR12	SADSCATCATYPSEIDT	GSYDSSYVGYVGV
AR13	SADSCATCATYPSEIDT	GSYDNTYAGI
AR14	SADSCATCATYPSEIDT	GGYDSSSGA
AR15	SADSCATCATYPSEIDT	GAYDSSYIGI

* AA: amino acid.

**Table 3:** Clinical usage of small molecule inhibitors targeting c-Met in cancer therapy.

Drug Name	Targets	FDA Approval Status	Approved Year
Tivantinib	c-Met, microtubule	None	N.A.*
Foretinib	c-Met, VEGFR-2 *	None	N.A.
Cabozantinib	c-Met, VEGFR, Axl	Medullary thyroid cancer Advanced renal cell carcinoma Hepatocellular carcinoma	2012 2016 2019
Crizotinib	c-Met, ALK *, ROS1, RON *	ALK or ROS-1 positive NSCLC *	2011
Capmatinib	c-Met, EGFR *, ErbB-3	None	N.A.
AMG337	c-Met	None	N.A.
AZD6094	c-Met	None	N.A.
BMS777607/ASLAN002	c-Met, Axl, Tyro3, RON	None	N.A.
Glesatinib	c-Met, Axl	None	N.A.
Tepotinib	c-Met	None	N.A.

* VEGFR-2: Vascular endothelial growth factor-2, ALK: Anaplastic lymphoma kinase, RON: Receptor d’Origine nantais, EGFR: Epidermal growth factor receptor, NSCLC: Non-small cell lung cancer, N.A.: not available.

## Supplementary Materials

The following are available online at https://www.mdpi.com/2218-273X/10/3/421/s1, Figure S1: Evaluation of 6 prediction models using training data sets, Figure S2: Shannon’s entropy (SE) change following biopanning procedure, Table S1: Accuracy score distributions of random forest (RF), regularized discriminant analysis (RDA), linear discriminant analysis (LDA), support vector machines (SVM), naïve bayes (NB), AdaBoost Classification Trees (ADA) for HCDR3 binding reactivity predictions, Table S2: Kappa score distributions of RF, RDA, LDA, SVM, NB, and ADA for HCDR3 binding reactivity predictions, Table S3: Accuracy score distributions of RF, RDA, LDA, SVM, NB, and ADA for LCDR3 binding reactivity predictions, Table S4: Kappa score distributions of RF, RDA, LDA, SVM, NB, and ADA for LCDR3 binding reactivity predictions, Table S5: Optimal parameter tuning in generation of random forest model, Table S6: Biopanning titer following four rounds of biopanning, Table S7: Mean-minimal depth of each variables and interaction (HCDR3), Table S8: Mean-minimal depth of each variables and interaction (LCDR3), Table S9: Predicted clones with HCDR3, full variable domain sequences with prediction results and confidence value, Table S10: Predicted clones with LCDR3, full variable domain sequences with prediction results and confidence value.
