# Supplementary material for: Machine Learning-Guided Prediction of Antigen-Reactive In Silico Clonotypes Based on Changes in Clonal Abundance through Bio-Panning
Source: Biomolecules. 2020 Mar 8;10(3):421. doi: 10.3390/biom10030421 (PMC7175295; doi:10.3390/biom10030421)
Supplement: Supplementary file 1 [file biomolecules-10-00421-s001.zip › biomolecules-697253-supplementary/Supplementary Materials.docx]

Supplementary Materials for

**Machine learning-guided prediction of antigen-reactive in silico clonotypes based on changes in clonal abundance through biopanning**

Duck Kyun Yoo^†^, Seung Ryul Lee^†^, Yushin Jung, Haejun Han, Hwa Kyoung Lee, Jerome Han, Soohyun Kim, Jisu Chae, Taehoon Ryu^*^ and Junho Chung^*^

Department of Biochemistry and Molecular Biology, Seoul National University College of Medicine, Seoul National University, Seoul 03080, Republic of Korea

^*^Corresponding authors: Taehoon Ryu and Junho Chung

**Email**: taehoon.ryu@celemics.com and junhochung@icloud.com

This file includes:

Figure S1. and S2.

Table S1. to Table S10.

**Table S1.** Accuracy score distributions of random forest (RF), regularized discriminant analysis (RDA), linear discriminant analysis (LDA), support vector machines (SVM), naïve bayes (NB), AdaBoost Classification Trees (ADA) for HCDR3 binding reactivity predictions.

| Models | **Min.** | **1^st^ Qu.** | **Median** | **Mean** | **3^rd^ Qu.** | **Max** |
| --- | --- | --- | --- | --- | --- | --- |
| RF | 0.8518519 | 0.8685360 | 0.8963675 | 0.8957117 | 0.9187228 | 0.9622642 |
| RDA | 0.8301887 | 0.8679245 | 0.8846154 | 0.8824303 | 0.9069706 | 0.9245283 |
| LDA | 0.8301887 | 0.8497554 | 0.8586182 | 0.8597816 | 0.8653846 | 0.9074074 |
| SVM | 0.8333333 | 0.8490566 | 0.8518519 | 0.8585793 | 0.8653846 | 0.8867925 |
| NB | 0.7735849 | 0.8301887 | 0.8476052 | 0.8473414 | 0.8862482 | 0.9056604 |
| ADA | 0.1320755 | 0.1346154 | 0.1481481 | 0.1433111 | 0.1502446 | 0.1509434 |

**Table S2.** Kappa score distributions of RF, RDA, LDA, SVM, NB, and ADA for HCDR3 binding reactivity predictions.

| Models | **Min.** | **1^st^ Qu.** | **Median** | **Mean** | **3^rd^ Qu.** | **Max** |
| --- | --- | --- | --- | --- | --- | --- |
| RF | 0.14960630 | 0.3677786 | 0.4269277 | 0.44620053 | 0.5648536 | 0.8354037 |
| RDA | -0.03470716 | 0.1952278 | 0.3065421 | 0.30886520 | 0.4883267 | 0.6293706 |
| LDA | -0.03470716 | 0.0000000 | 0.0000000 | 0.04000627 | 0.0000000 | 0.5054945 |
| SVM | -0.03404255 | 0.0000000 | 0.0000000 | 0.02684002 | 0.0000000 | 0.2243902 |
| NB | 0.01547988 | 0.1338836 | 0.3016105 | 0.29064642 | 0.4346762 | 0.6120059 |
| ADA | 0.00000000 | 0.00000000 | 0.00000000 | 0.00000000 | 0.00000000 | 0.00000000 |

**Table S3.** Accuracy score distributions of RF, RDA, LDA, SVM, NB, and ADA for LCDR3 binding reactivity predictions.

| Models | **Min.** | **1^st^ Qu.** | **Median** | **Mean** | **3^rd^ Qu.** | **Max** |
| --- | --- | --- | --- | --- | --- | --- |
| RDA | 0.8269231 | 0.8461538 | 0.8762700 | 0.8724923 | 0.8981687 | 0.9245283 |
| RF | 0.8039216 | 0.8461538 | 0.8490566 | 0.8572842 | 0.8679245 | 0.9433962 |
| LDA | 0.7692308 | 0.7884615 | 0.8039216 | 0.8049693 | 0.8269231 | 0.8461538 |
| NB | 0.7500000 | 0.7762671 | 0.8058069 | 0.8023454 | 0.8254717 | 0.8653846 |
| SVM | 0.7692308 | 0.7692308 | 0.7735849 | 0.7743969 | 0.7735849 | 0.7884615 |
| ADA | 0.1153846 | 0.1698113 | 0.1808781 | 0.1829930 | 0.2105406 | 0.2307692 |

**Table S4.** Kappa score distributions of RF, RDA, LDA, SVM, NB, and ADA for LCDR3 binding reactivity predictions.

| Models | **Min.** | **1^st^ Qu.** | **Median** | **Mean** | **3^rd^ Qu.** | **Max** |
| --- | --- | --- | --- | --- | --- | --- |
| RDA | 0.3389831 | 0.4765432 | 0.5962299 | 0.5791726 | 0.67546043 | 0.7557604 |
| RF | 0.2145015 | 0.4825452 | 0.5323955 | 0.5351669 | 0.58639911 | 0.8335079 |
| LDA | 0.0000000 | 0.1226994 | 0.1355932 | 0.1953858 | 0.25949367 | 0.4347826 |
| NB | 0.0000000 | 0.1569665 | 0.2358027 | 0.2448634 | 0.34024896 | 0.5235602 |
| SVM | 0.0000000 | 0.0000000 | 0.0000000 | 0.0000000 | 0.0000000 | 0.0000000 |
| ADA | -0.2510460 | -0.1787248 | -0.1411647 | -0.1307576 | -0.07048755 | 0.0000000 |


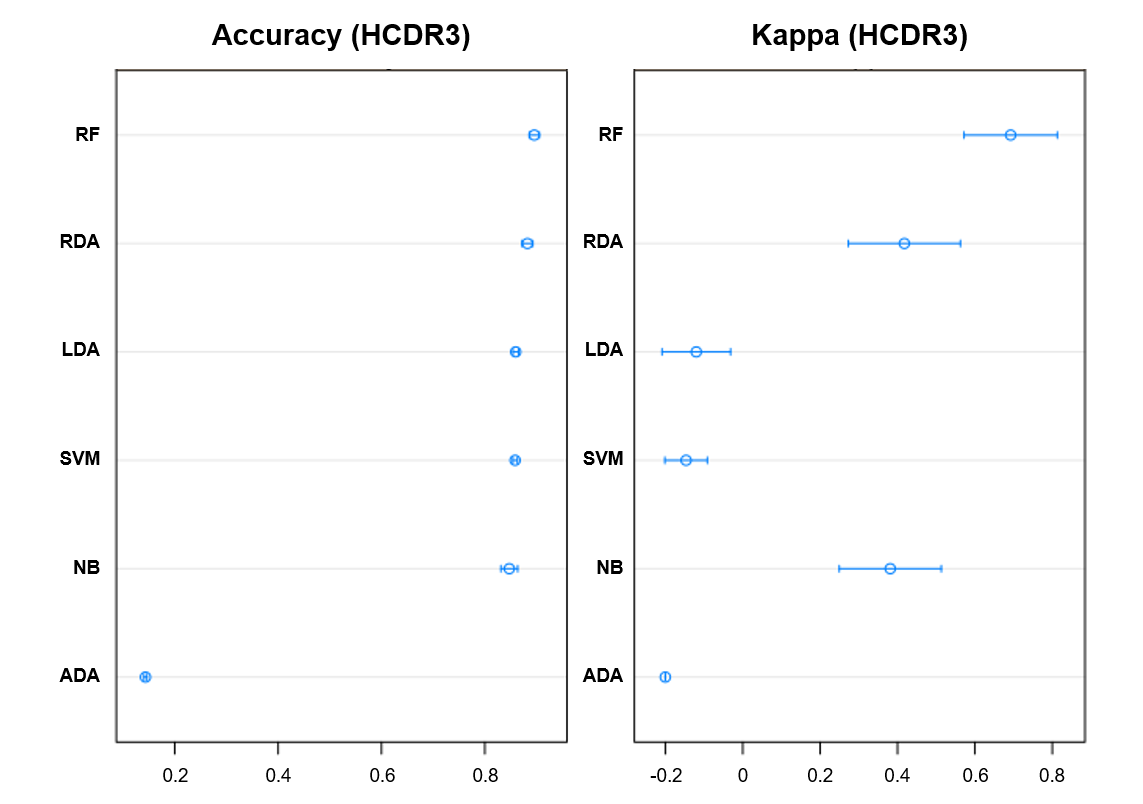

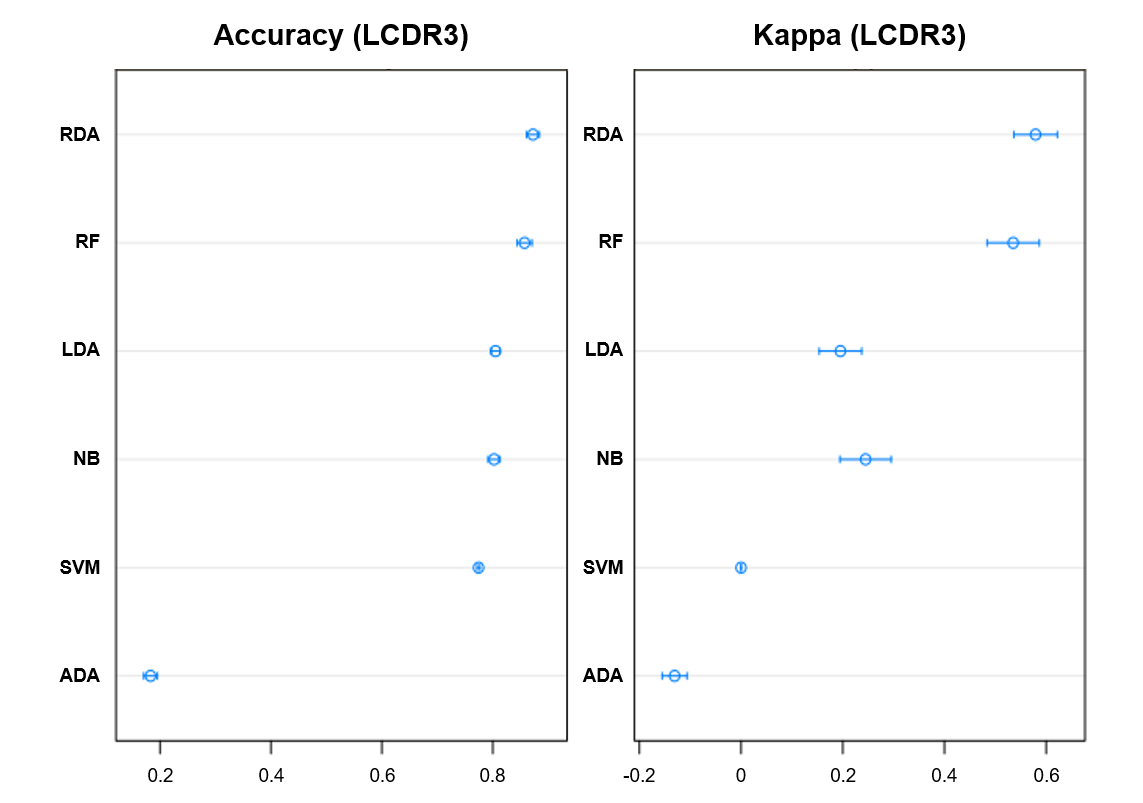


Accuracy: HCDR3

Cohen’s Kappa: HCDR3

Accuracy: LCDR3

Cohen’s kappa: LCDR3

**Figure S1.** Evaluation of 6 prediction models using training data sets. RF: random forest, RDA: regularized discriminant analysis, LDA: linear discriminant analysis, NB: naïve bayes, SVM: support vector machine, ADA: AdaBoost classification trees. Accuracy and Cohen’s kappa value were calculated and plotted as an evaluation metric.

**Table S5.** Optimal parameter tuning in generation of random forest model.

table is attached as separate file (Table S5.xlsx)

**Table S6.** Biopanning titer following four rounds of biopanning.

|  | 0.05% PBST  wash | **antigen: mouse c-Met** | |
| --- | --- | --- | --- |
|  |  | input titer (PFU/mL) | output titer (PFU/mL) |
| Round 0 | - | **4.96 x 10^9^** | |
| Round 1 | x 1 | **2.44 x 10^11^** | **6.26 x 10^7^** |
| Round 2 | x 3 | **3.28 x 10^11^** | **8.80 x 10^4^** |
| Round 3 | x 3 | **2.51 x 10^12^** | **1.12 x 10^7^** |
| Round 4 | x 5 | **2.68 x 10^11^** | **1.24 x 10^7^** |

**Figure S2.** Shannon’s entropy (SE) change following biopanning procedure. Yellow and blue dots represent SE of LCDR3 and HCDR3, respectively.

**Table S7.** Mean-minimal depth of each variables and interaction (HCDR3).

| **root_variable** | **variable** | **mean_min_depth** | **occurrences** | **interaction** |
| --- | --- | --- | --- | --- |
| HCDR3.R3 | HCDR3.R1 | 0.836608 | 470 | HCDR3.R3:HCDR3.R1. |
| HCDR3.R3 | HCDR3.R0 | 1.232051 | 460 | HCDR3.R3:HCDR3.R0. |
| HCDR3.R3 | HCDR3.R4 | 1.381857 | 474 | HCDR3.R3:HCDR3.R4. |
| HCDR3.R3 | HCDR3.R2 | 1.489798 | 471 | HCDR3.R3:HCDR3.R2. |
| HCDR3.R3 | HCDR3.R3 | 1.554127 | 462 | HCDR3.R3:HCDR3.R3. |
| HCDR3.R0 | HCDR3.R3 | 1.873671 | 379 | HCDR0.R3:HCDR3.R3. |
| HCDR3.R4 | HCDR3.R4 | 1.922962 | 416 | HCDR3.R4:HCDR3.R4. |
| HCDR3.R1 | HCDR3.R3 | 1.952568 | 399 | HCDR3.R1:HCDR3.R3. |
| HCDR3.R1 | HCDR3.R4 | 2.054730 | 407 | HCDR3.R1:HCDR3.R4. |
| HCDR3.R4 | HCDR3.R3 | 2.058536 | 397 | HCDR3.R4:HCDR3.R3. |
| HCDR3.R4 | HCDR3.R2 | 2.191051 | 403 | HCDR3.R4:HCDR3.R2. |
| HCDR3.R1 | HCDR3.R3 | 2.335815 | 402 | HCDR3.R1:HCDR3.R3. |
| HCDR3.R0 | HCDR3.R4 | 2.371460 | 367 | HCDR3.R0:HCDR3.R4. |
| HCDR3.R1 | HCDR3.R1 | 2.412306 | 388 | HCDR3.R1:HCDR3.R1. |
| HCDR3.R1 | HCDR3.R2 | 2.414817 | 401 | HCDR3.R1:HCDR3.R2. |
| HCDR3.R0 | HCDR3.R2 | 2.527646 | 375 | HCDR3.R0:HCDR3.R2. |
| HCDR3.R2 | HCDR3.R4 | 2.534346 | 296 | HCDR3.R2:HCDR3.R4. |

**Table S8.** Mean-minimal depth of each variables and interaction (LCDR3).

| **root_variable** | **variable** | **mean_min_depth** | **occurrences** | **interaction** |
| --- | --- | --- | --- | --- |
| LCDR3.R4 | LCDR3.R0 | 1.180377 | 444 | LCDR3.R4:LCDR3.R0 |
| LCDR3.R4 | LCDR3.R2 | 1.428251 | 446 | LCDR3.R4:LCDR3.R2 |
| LCDR3.R3 | LCDR3.R0 | 1.465202 | 426 | LCDR3.R3:LCDR3.R0 |
| LCDR3.R4 | LCDR3.R1 | 1.486852 | 432 | LCDR3.R4:LCDR3.R1 |
| LCDR3.R4 | LCDR3.R3 | 1.549865 | 436 | LCDR3.R4:LCDR3.R3 |
| LCDR3.R3 | LCDR3.R1 | 1.555740 | 431 | LCDR3.R3:LCDR3.R1 |
| LCDR3.R3 | LCDR3.R4 | 1.632188 | 427 | LCDR3.R3:LCDR3.R4 |
| LCDR3.R4 | LCDR3.R4 | 1.633327 | 435 | LCDR3.R4:LCDR3.R4 |
| LCDR3.R3 | LCDR3.R3 | 1.720592 | 430 | LCDR3.R3:LCDR3.R3 |
| LCDR3.R0 | LCDR3.R2 | 1.785722 | 428 | LCDR3.R3:LCDR3.R2 |
| LCDR3.R1 | LCDR3.R4 | 1.932578 | 391 | LCDR3.R0:LCDR3.R4 |
| LCDR3.R1 | LCDR3.R4 | 1.944619 | 385 | LCDR3.R1:LCDR3.R4 |
| LCDR3.R0 | LCDR3.R3 | 2.245964 | 377 | LCDR3.R1:LCDR3.R3 |
| LCDR3.R2 | LCDR3.R3 | 2.255933 | 375 | LCDR3.R0:LCDR3.R3 |
| LCDR3.R2 | LCDR3.R3 | 2.256614 | 371 | LCDR3.R2:LCDR3.R3 |
| LCDR3.R2 | LCDR3.R1 | 2.276000 | 370 | LCDR3.R2:LCDR3.R1 |
| LCDR3.R2 | LCDR3.R4 | 2.279314 | 377 | LCDR3.R2:LCDR3.R4 |

**Table S9.** Predicted clones with HCDR3, full variable domain sequences with prediction results and confidence value

| **Clone ID** | **Selected HCDR3** | **Mapped VH** | **Prediction** | **Probability** |
| --- | --- | --- | --- | --- |
| RFAR1 | SAGIGGDCIDA | AVTLDESGGGLQTPGGTLSLVCKASGFTFSSYNMGWVRQAPGKGLEWVAAISNDGSSTGYATAVKG  RATISRDNGQSTVRLQLNNLRAEDTGTYYCAKSAGIGGDCIDAWGHGTEVIVSS | AR | 0.99 |
| RFAR2 | CADTGYGCAYCIDA | AVTLDESGGGLQTPGGTLSLVCKASGFTFSSFNMFWVRQAPGKGLEFVASISNTGSYTKYGAAVKG  RATISRDDGQSTVRLQLNNLRAEDTGTYYCTRCADTGYGCAYCIDAWGHGTEVIVSS | AR | 0.99 |
| RFAR3 | TAGTCTTSCNAGAYIDA | AVTLDESGGGLQTPGGALSLVCKASGFTFSSFNMFWVRQAPGKGLEFVASISNTGSTTGYGPAVKG  RATISRDDGQSTVRLQLNNLRAEDTATYFCAKTAGTCTTSCNAGAYIDAWGHGTEVIVSS | AR | 0.96 |
| RFAR4 | AVGFACGWCSAGIDA | AVTLDESGGGLQTPGGTLSLVCKASGFSFSSFYMFWVRQAPGKGLEFVAQISSTGSSTDYGSAVKG  RATISRDNGQSTLRLQLNNLRAEDTGTYFCAKAVGFACGWCSAGIDAWGHGTEVIVSS | AR | 0.96 |
| RFAR5 | SADSCATCATYPSEIDT | AVTLDESGGGLQTPGGGLSLVCKASGFTFTDYGMGWMRQAPGKGLEYVAGISNDGSSVAYGSAVKG  RATISRDNGQSTVRLQLNNLRAEDTGTYYCARSADSCATCATYPSEIDTWGHGTEVIVSS | AR | 0.95 |
| RFAR6 | SGSNWWADSTGNVDA | AVTLDESGGGLQTPGGALSLVCKASGFTFNNYAMNWVRQAPGKGLEYVAAISSSASYTNYGAAVKG  RATISRDNGQSTVRLQLNNLRAEDTATYYCAKSGSNWWADSTGNVDAWGHGTEVIVSS | AR | 0.95 |
| RFAR7 | SPGGYCCAGWIDA | AVTLDESGGGLQTPGGGLSLVCKASGFTFSSYNMGWVRQAPGKGLEWVAGIYSGNRTYYAPAVKG  RATISRDNGQSTVRLQLNNLRAEDTATYFCARSPGGYCCAGWIDAWGHGTEVIVSS | AR | 0.95 |
| RFAR8 | SPGAFTYVSGIDA | AVTLDESGGGLQTPGGALSLVCKASGFTFSDYDMAWVRQAPGKGLEFVAGITSDGSNTGYGSAVKG  RATISRDNGQSSVRLQLNNLRAEDTGTYICARSPGAFTYVSGIDAWGHGTEVIVSS | AR | 0.95 |
| RFAR9 | SVTGCGGDYAWCAFGDLDHIDA | AVTLDESGGGLQTPGRALSLVCKASGFTFSSFNMFWVRQAPGKGLEYVAAISSTGSYTKYGAAVQG  RATISRDNGQSTVRLQLNNLRAEDTSTYFCAKSVTGCGGDYAWCAFGDLDHIDAWGHGTEVIVSS | AR | 0.95 |
| RFAR10 | ASGGGYCSWGACIVAWIGT | AVTLDESGGGLQTPGGTLSLVCKASGFSISSYGMGWMRQAPGKGLEFVASISNTGSYTNYGSAVKG  RATISRDNGQSTVRLQLNNLRAEDTATYYCAKASGGGYCSWGACIVAWIGTWGHGTEVIVSS | AR | 0.95 |
| RFAR11 | TTVISCGTLCAGHIDA | AVTLDESGGGLQTPGGTLSLVCKASGFSFSSFYMFWVRQAPGKGLEFVAQISNTGSSTDYGSAVKG  RATISRDNGQSTVRLQLNNLRAEDTAIYYCAKTTVISCGTLCAGHIDAWGHGTEVIVSS | AR | 0.95 |
| RFAR12 | GASSGSGCAGGLCAGEIDA | AVTLDESGGGLQTPGGTLSLVCKGSGFTFSSVNMGWMRQAPGKGLEWVADINSAGSSTNYGAAVKG  RATISRDNGQSTVRLQLNNLRAEDTGIYFCAKGASSGSGCAGGLCAGEIDAWGHGTEVIVSS | AR | 0.95 |
| RFAR13 | GSGGVDSIDA | AVTLDESGGGLQTPGGAFSLVCKGSGFTFSSFNMFWVRQAPGKGLEYVAGIYYSGSGTGNGAAVKG  RATISRDNGQSTVRLQLNNLRAEDTGTYYCARGSGGVDSIDAWGHGTEVIVSS | AR | 0.95 |
| RFAR14 | TADDGNCCGGDNIDA | AVTLDESGGGLQTPGGGLSLVCKASGFTFSDYGMGWVRQAPGKGLEWVAGIYTGSYTGYGSAVKG  RATISRDNGQSTVRLQLNNLRAEDTGTYYCAKTADDGNCCGGDNIDAWGHGTEVIVSS | AR | 0.95 |
| RFAR15 | AYSGGFYCAGSLCAAHAGLIDA | AVTLDESGGGLQTPGGALSLVCKASGFTFSSYGMFWVRQAPGKGLEWIAGISNSGSYTAYGAVDG  RATISRDNGQSTLRLQLNNLRAEDTATYYCAKAYSGGFYCAGSLCAAHAGLIDAWGHGTEVIVSS | AR | 0.95 |

(continued)

| **Clone ID** | **Selected HCDR3** | **Mapped VH** | **Prediction** | **Probability** |
| --- | --- | --- | --- | --- |
| RFAR16 | AAASGCAGDNIDA | AVTLDESGGGLQTPGGALSLVCKASGFTFSDYGMGWMRQAPGKGLEFVAGIGNTGSWTAYGAAVKG  RATISRDNGQSTVRLQLNNLRAEDTATYYCAKAAASGCAGDNIDAWGHGTEVIVSS | AR | 0.95 |
| RFAR17 | STSDYGGWYGADLDSIDA | AVTLDESGGGLQTPGGALSLVCKASGFTFSSFNMFWVRQAPGKGLEWVAQISGDGSTYYAPAVQG  RATISRDNGQSTVRLQLNNLRAEDTGTYFCAKSTSDYGGWYGADLDSIDAWGHGTEVIVSS | AR | 0.95 |
| RFAR18 | TADGGWFGNSAGSIDA | AVTLDESGGGLQTPGGTLSLVCKASGFSISSYTMQWVRQAPGKGLEWVAGISSSGRYTDYGAAVKG  RATISRDNGQSTVRLQLNNLRAEDTGIYFCAKTADGGWFGNSAGSIDAWGHGTEVIVSS | AR | 0.95 |
| RFAR19 | TSGYCGWCGAYNIDA | AVTLDESGGGLQTPGGALSLVCKASGFTFSSFNMFWVRQAPGKGLEYVAEISSTGSWTGYGSAVKG  RATISRDNGQSTVRLQLNNLRAEDTGTYYCAKTSGYCGWCGAYNIDAWGHGTEVIVSS | AR | 0.95 |
| RFAR20 | SANSGRSASQMDA | AVTLDESEGGLQTPGGALSLVCKASGFTFSDYAMGWVRQAPGKGLEYVASIRGAGSSDTSYGAAVKG  RATISRDNGQSTVRLQLNNLRAEDTGTYYCAKSANSGRSASQMDAWGHGTEVIVSS | AR | 0.95 |
| RFAR21 | GGSGYCGWSGYSCVGEIDA | AVTLDESGGGLQTPGGTLSLVCKASGFTFSSSYGMHWVRQAPGKGLEWVAGIYSGGGNTYYAPAVKG  RATISRDNGQSTVRLQLNDLRAEDTATYYCTRGGSGYCGWSGYSCVGEIDAWGHGTEVIVSS | AR | 0.95 |
| RFAR22 | ATGTGYYGSDSYVSSIDA | AVTLDESGGGLQTPGGTLSLVCKGSGFTFSSYDMYWVRQAPGKGLEYVAVISSDGRYTNYGSAVKG  RATISKDNGQSTVRLQLNNLRAEDTGTYYCAKATGTGYYGSDSYVSSIDAWGHGTEVIVSS | AR | 0.95 |
| RFAR23 | SDISWCAWCATDLGQIDA | AVTLDESGGGLQTPGGTLSLVCKASGFTFSSFNMFWVRQAPGKGLEYVASISSADIWTGYGSAVKG  RATISRDDGQSTVRLQLNNLRAEDTGTYYCAKSDISWCAWCATDLGQIDAWGHGTEVIVSS | AR | 0.95 |
| RFAR24 | GAYGHCSGSWCSAGLIDA | AVTLDESGGGLQTPGGTLSLVCKASGFNFSSYQMNWIRQAPGKGLEFVAAINRFGNSTGYAAAVKG  RATISRDDGQSTVRLQLNNLRAEDTGTYYCAKGAYGHCSGSWCSAGLIDAWGHGTEVIVSS | AR | 0.95 |
| RFAR25 | DVYGWCASDCGGSDTIDA | AVTLDESGGGLQTPGGALSLVCKASGFSISSYGMFWVRQAPGKGLEFVAGISSSGRHTDYGSAVKG  RATISRDNGQSTMRLQLNNLRAEDTGTYFCAKDVYGWCASDCGGSDTIDAWGHGTEVIVSS | AR | 0.95 |
| RFAR26 | SAAGYGCTYGSGYGWCVNYIDA | AVTLDESGGGLQTPGRALSLVCKASGFTFSSFNMFWVRQAPGKGLEFVAAISSSGRYTGYGSAVKG  RATISRDNGQSTVRLQLNNLRAEDTAIYFCAKSAAGYGCTYGSGYGWCVNYIDAWGHGTEVIVSS | AR | 0.95 |
| RFAR27 | AAACSGNDCAALLAAGIDA | AVTLDESGGGLQTPGGTLSLVCKASGFTFSSYAMNWVRQAPGKGLEWVGVISDSGNTPKYGPAVKG  RATISRDNGQSTVRLQLNNLRAEDTGTYYCAKAAACSGNDCAALLAAGIDAWGHGTEVIVSS | AR | 0.95 |
| RFAR28 | DDSSCIWNTGCTGLIDE | AVTLDESGGGLQTPGGTLSLVCKGSGFTFSSVNMFWVRQAPGKGLEWVAEISTTGRYTNYGSAVKG  RATISRDNGQSTVRLQLNNLRAEDTGTYYCAKDDSSCIWNTGCTGLIDEWGHGTEVIVSS | AR | 0.95 |
| RFAR29 | SADGYGWDTAGNMDA | AVTLDESGGGLQTPGGGLSLVCKASGFTFSSNAMGWMRQAPSKGLEFVAAISSSGSGTYYGAAVKG  RATISRDDGQSTVRLQLNNLRAEDTAIYFCAKSADGYGWDTAGNMDAWGHGTEVIVSS | AR | 0.95 |
| RFAR30 | SGTGKYTTGQIDA | AVTLDESGGGLQTPGGTLSLVCKGSGFTFSSFNMFWVRQAPGKGLEYVAEITSGGSYTYYGAAVKG  RATISRDNGQSTVRLQLNNLRAEDTGTYYCARSGTGKYTTGQIDAWGHGTEVIVSS | AR | 0.95 |

(continued)

| **Clone ID** | **Selected HCDR3** | **Mapped VH** | **Prediction** | **Probability** |
| --- | --- | --- | --- | --- |
| RFAR31 | TTDSAYCCAGEIDT | AVTLDESGGGLQTPGGTLSLVCKASGFTFSSYGMNWVRQAPGKGLEYVAAISSTGTTTNYGSAVKG  RATISRDNGQSTVRLQLNNLRAEDTGIYYCAKTTDSAYCCAGEIDTWGHGTEVIVSS | AR | 0.95 |
| RFAR32 | TATTCTGCWAGIDSIDA | AVTLDESGGGLQTPGRALSLVCKASGFTFNTYTMFWVRQAPGKGLEFVAGIDNTGSSTGYGPAVQG  RATISRDNGQSTVRLQLNNLRAEDTATYYCAKTATTCTGCWAGIDSIDAWGHGTEVIVSS | AR | 0.95 |
| RFAR33 | SAADYTCGNGGGSCAGSIDA | AVTLDESGGGLQTPGRALSLVCKASGFTFNTYTMFWVRQAPGKGLEWVAQTSNTGRYTAYGPAVKG  RATISRDNGQSTVRLQLNNLRAEDTGIYYCAKSAADYTCGNGGGSCAGSIDAWGHGTEVIVSS | AR | 0.95 |
| RFAR34 | TTGSDYCTLCTGGIDA | AVTLDESGGGLQTPGGGLSLVCKASGFSFSSYDMLWVRQAPGKGLEFVGVISSSGRYTSYGAAVKG  RATISRDNGQSTVRLQLNNLRAEDTGTYYCAKTTGSDYCTLCTGGIDAWGRGTEVIVSS | AR | 0.95 |
| RFAR35 | GGGSDSCTACAGSIDA | AVTLDESGGGLQTPGGGLSLICKASGFTFSDYGMGWMRQAPGKGLEYVGVISSSGSTTRYGSAVKG  RATISRDNGQSTVRLQLNNLRAEDTGIYYCTRGGGSDSCTACAGSIDAWGHGTEVIVSS | AR | 0.95 |
| RFAR36 | AAGDSGYAGRIDA | AVTLDESGGGLQTPGGALSLVCKASGFTFSSFYMFWVRQAPGKGLEYVAQISGDGSWTYYGSAVKG  RATISRDNGQSTVRLQLNNLRAEDTGIYYCAKAAGDSGYAGRIDAWGHGTEVIVSS | AR | 0.95 |
| RFAR37 | TTCSGSYGWCADSIDA | AVTLDESGGGLQTPGGGLSLVCKASGFTISDYGMGWVRQAPGKGLEYVAQINSAGSYPKYGAAVKG  RATISKDNGQSTVRLQLNNLRAEDTATYYCAKTTCSGSYGWCADSIDAWGHGTEVIVSS | AR | 0.95 |
| RFAR38 | SATTGGAWAGEIDT | AVTLDESGGGLQTPGGGLSLVCKASGFTFSDYQMNWIRQAPGKGLEWVAGISSGGGYTYYGSAVKG  RATISRDNGQSTVRLQLNNLRAEDTGIYFCGKSATTGGAWAGEIDTWGHGTEVIVSS | AR | 0.95 |
| RFAR39 | GCAGCGWSAARIDA | AVTLDESGGGLQTPGGALSLVCKGSGFTFSSYAMFWVRQEPGKGLECVGYINNDGSSTWYATAVKG  RATISRDNGQSTVRLQLNNLRAEDTATYYCARGCAGCGWSAARIDAWGHGTEVIVSS | AR | 0.95 |
| RFAR40 | DTNRDCHSDADSIDA | AVTLDESGGGLQTPGGALSLVCKASGFTFSSYAMNWVRQAPGKGLEWVGGIGSTGSGTYYAPAVQG  RATISRDNGQSTVRLQLNNLRAEDTGTYYCAKDTNRDCHSDADSIDAWGHGTEVIVSS | AR | 0.95 |
| RFNR1 | DAYGYNGWRAGSIDA | AVTLDESGGGLQTPGGTLSLVCKGSGFTFSSVNMAWVRQAPGKGLEFVAEISSDAGSWTAYGAAVKG  RATISRDNGQSTVRLQLNNLRAEDTGTYFCAKDAYGYNGWRAGSIDAWGHGTEVIVSS | NR | 0.00 |
| RFNR2 | NSGSGGWITDTGRIDA | AVTLDESGGGLQMPGGALSLVCKASGFTFSSYEMQWVRQAPGKGLEWVAGIYSGGTTTSYGPAVKG  RATISRDDGQSTVRLQLNNLRAEDTGTYYCAKNSGSGGWITDTGRIDAWGHGTEVIVSS | NR | 0.00 |
| RFNR3 | SADNGWNTAGRIDA | AVTLDESGGGLQTPGGTLSLICKASGFTFSSVNMGWVRQAPGKGLEFIAQITSRGSSTYYAPAVKG  RATISRDNGQSTVRLQLNNLRAEDTGTYYCARSADNGWNTAGRIDAWGHGTEVIVSS | NR | 0.00 |
| RFNR4 | AAGSGTGWSAGGIDA | AVTLDESGGGLQTPGGALSLVCKGSGFTFNSYAMQWVRQAPGKGLEWVAGISGSGSYTAYGAAVKG  RATISRDNGQSTVRLQLNNLRAEDTATYYCAKAAGSGTGWSAGGIDAWGHGTEVIVSS | NR | 0.00 |
| RFNR5 | SGDAATPDAGGIDA | AVTLDESGGGLQTPGGGLSLVCKGSGFTFSSFNMFWVRQAPGKGLEFVAAINSGGRYTGYGSAVKG  RATISRDNGQSTVRLQLNNLRAEDTGIYYCARSGDAATPDAGGIDAWGHGTEVIVSS | NR | 0.00 |
| RFNR6 | SGYGGYDGSNIDA | AVTLDESGGGLQTPGGGLSLVCKASGFTFSSHGMGWVRQAPGKGLEWVAGIYSGGRYTYYGAAVKG  RATISRDNGQSTVRLQLNNLRAEDTAIYYCAKSGYGGYDGSNIDAWGHGTEVIVSS | NR | 0.00 |
| RFNR7 | ATYAGSGCCDNIDA | AVTLDESGGGLQTPGGVLSLVCKASGFDFSNNDMAWVRQAPGKGLEFVADISSGGGSYTYYGSAVKG  RATISRDNGQSTVRLQLNNLRAEDTATYFCARATYAGSGCCDNIDAWGHGTEVIVSS | NR | 0.00 |
| RFNR8 | GACGGGCYTATFIGTIDV | AVTLDESGGGLQTPGGTLSLVCKGSGFTFSSVNMGWMRQAPGKGLEYVAEISGSGSWTYYAPAVKG  RATISRDNGQSTVRLQLNNLRAEDTGTYFCAKGACGGGCYTATFIGTIDVWGHGTEVIVSS | NR | 0.00 |
| RFNR9 | SAAGYGCAYGWCGDSIDA | AVTLDESGGGLQTPGGALSLVCKASGFSISSYDMAWVRQAPGKGLEFVAGIYSGTTTAYGAAVKG  RATISRDDGQSTVRLQLNNLRAEDTATYYCAKSAAGYGCAYGWCGDSIDAWGHGTEVIVSS | NR | 0.00 |
| RFNR10 | AAGTCYGCSFYATNIDA | AVTLDESGGGLQTPGGALSLVCKGSGFTFSSVNMFWVRQAPGKGLEWVAGIDNTGRYTSYGSAVKG  RATISRDNGQSTVRLQLNNLRAEDTAIYFCAKAAGTCYGCSFYATNIDAWGHGTEVIVSS | NR | 0.00 |

**Table S10.** Predicted clones with LCDR3, full variable domain sequences with prediction results and confidence value

| **Clone ID** | **Selected LCDR3** | **Mapped V_L_** | **Prediction** | **Probability** |
| --- | --- | --- | --- | --- |
| RFAR1 | GNYDGSSSVGI | LTQPSSVSANLGGTVKITCSGGSGDYGWYQQKSPGSAPVTVIYWDDERPSGIPS  RFSGSTSGSTNTLTITGVQADDEAVYFCGNYDGSSSVGIFGAGTTLTVL | AR | 0.99 |
| RFAR2 | GSRDSTLAA | LTQPSSVSANLGGTVEITCSGGSGSYGWYQQKSPGSAPVTVIYYNTNRPSDIPS  RFSGSKSGSTGTLTITGVQAEDEAVYFCGSRDSTLAAFGAGTTLTVL | AR | 0.99 |
| RFAR3 | GSYDSSYVGYVGV | LTQPSSVSANLGGTVEITCSGGSGSYGWYQQKSPGSAPVTVIYNDNQRPSNIPS  RFSGALSGSTATLTITGVQAEDEAVYYCGSYDSSYVGYVGVFGAGTTLTVL | AR | 0.99 |
| RFAR4 | GNKDN | LTQPSSVSANPGGTVEITCSGGSGSYGWFQQKAPGSAPVTLIYANTNRPSDIPS  RFSGSKSGSTNTLTITGVQADDEAVYYCGNKDNFGAGTTLTVL | AR | 0.99 |
| RFAR5 | GGYDSTYAGL | LTQPSSVSANLGGTVEITCSGGSYYGWYQQKSPGSAPVTLIYNNDKRPSDIPS  RFSGSKSGSTGTLTITGVRAEDEAVYYCGGYDSTYAGLFGAGTTLTVL | AR | 0.99 |
| RFAR6 | GTADSSGTV | LTQPSSVSANPGETVKITCSGGGSSSYYGWYQQKSPGSAPVTLIYESNKRPSDIPS  RFSGSKSGSTATLTITGVQADDEAVYYCGTADSSGTVFGAGTTLTVL | AR | 0.99 |
| RFAR7 | GSRDSSYVPI | LTQPSSVSANLGGTVEITCSGGSGSYGWYQQKSPGSAPVTVIYYNTNRPSDIPS  RFSGSKSGSTHTLTITGVRAEDEAVYFCGSRDSSYVPIFGAGTTLTVL | AR | 0.99 |
| RFAR8 | GSWDSSSEGDSGYAGI | LTQPSSVSANPGETVKITCSGSRNSYGWYQQKSPGSAPVTVIYWNSNRPSGIPS  RFSGSTSGSTGTLTITGVQADDEAVYYCGSWDSSSEGDSGYAGIFGAGTTLTVL | AR | 0.99 |
| RFAR9 | GAYDSSYIGI | LTQPSSVSANLGGTVKITCSGGSSGYGWYQQKSPGSAPVTVIYSNTNRPSDIPS  RFSGSKSGSTGTLTITGVQAEDEAVYYCGAYDSSYIGIFGAGTTLTVL | AR | 0.99 |
| RFAR10 | GSFDSSYVGM | LTQPSSVSANPGETVKITCSGGSGNYGWYQQKSPGSAPVTVIYDSSSRPSDIPS  RFSGSTSGSTSTLTITGVQADDEAVYYCGSFDSSYVGMFGAGTTLTVL | AR | 0.99 |
| RFAR11 | GSIDSNYDGI | LTQPSSVSANPGETVKLICSGSSGDYGWYQQKSPGSAPVTVIYDNTNRPSNIPS  RFSGSLSGSTNTLSITGVQVEDEAVYFCGSIDSNYDGIFGAGTTLTVL | AR | 0.99 |
| RFAR12 | GSRDNSSAST | LTQPSSVSANPGETVEITCSGSSSGYGYGWYQQKSPGSAPVTLIYSNDKRPSDIPS  RFSGSKSGSTGTLTITGVRAEDEAVYFCGSRDNSSASTFGAGTTLTVL | AR | 0.99 |
| RFAR13 | GSFDSSSDSGYVGI | LTQPSSVSANPGETVKITCSGGSNNYGWYQQKSPGSAPVTVIYDNTNRPSDIPS  RFSGSASGSASTLTITGVQADDEAVYYCGSFDSSSDSGYVGIFGAGTALTVL | AR | 0.99 |
| RFAR14 | GSYDSSYVGL | LTQPSSVSANLGGTVEITCSGGSSNEYGWYQQKAPGSAPVTLIYDNTNRPSDIPS  RFSGSKSGSTGTLTIAGVQAEDEAVYFCGSYDSSYVGLFGAGTTLTVL | AR | 0.98 |
| RFAR15 | GSTDSSNTDI | LTQPSSVSAKPGGTVEITCSGGSGSYGWFQQKSPGSAPVTLIYANTNRPSDIPS  RFSGSKSGSTATLTITGVQAEDEAIYYCGSTDSSNTDIFGAGTTLTVL | AR | 0.98 |

(continued)

| **Clone ID** | **Selected LCDR3** | **Mapped V_L_** | **Prediction** | **Probability** |
| --- | --- | --- | --- | --- |
| RFAR16 | GSRAGSSI | LTQPSSVSANPGETVKITCSGSSGSYGWYQQKSPGSAPVTVIYYNDKRPSDIPS  RFSGSKSGSTGTLTITGVQAEDEAVYFCGSRAGSSIFGAGTTLTVL | AR | 0.98 |
| RFAR17 | GSYDSSYDGV | LTQPSSVSANPGETVKITCSGSSGYGYGWYQQKSPGSAPVTVIYYNDKRPSNIPS  RFSGSKSGSTATLTITGVRADDEAVYFCGSYDSSYDGVFGAGTTLTVL | AR | 0.98 |
| RFAR18 | GNGDRSSTTGI | LTQPSSVSANLGETVKITCSGGSGSYGWFQQKSPGSAPVTVIYSNDKRPSDIPS  RFSGSKSGSTGTLTITGVQADDEAVYYCGNGDRSSTTGIFGAGTTLTVL | AR | 0.97 |
| RFAR19 | GNEDISGI | LTQPSSVSANPGETVKITCSGGSYKYGWFQQKSPGSAPVTVIYYNDKRPSNIPS  RFSGSKSGSTATLTITGVQADDEAVYYCGNEDISGIFGAGTSLTVL | AR | 0.97 |
| RFAR20 | GSFDSSYTGI | LTQPSSVSANLGGTVKITCSGSSGSYGYGWYQQKSPGSAPVTVIYSNNQRPSNIPS  RFSGSTSGSTGTLTITGVRAEDEAVYYCGSFDSSYTGIFGAGTTLTVL | AR | 0.97 |
| RFAR21 | GSTDSSRTDT | LTQPSSVSANLGGTVKITCSGSSGSYGWYQQKSPGSAPVTLIYQNTKRPSDIPS  RFSGSKSGSTGTLTITGVQAEDEAVYYCGSTDSSRTDTFGAGTTLTVL | AR | 0.97 |
| RFAR22 | GSIDSRYVGI | LTQPSSVSANLGETVKITCSGGSYSYGWYQQKAPGSAPVTLIYDNTNRPSDIPS  RFSGSKSGSTHTLTITGVQADDEAVYFCGSIDSRYVGIFGAGTTLTVL | AR | 0.97 |
| RFAR23 | GGYDGSSAA | LTQPSSVSANPGGTVEITCSGGSGNNYGWFQQKSPGSTPVTVIYNNDKRPSDIPS  RFSGSKSGSTATLTITGVQADDEAVYYCGGYDGSSAAFGAGTTLTVL | AR | 0.97 |
| RFAR24 | ANYDSSTDI | LTQPSSVSANPGETVKITCSGGSSGYGYGWFQQKSPGSAPVTLIYYNDKRPSDIPS  RFSGSTSGSTSTLTITGVQADDEAVYYCANYDSSTDIFGAGTTLTVL | AR | 0.97 |
| RFAR25 | GSYDSTYAGM | LTQPSSVSANPGETVKITCSGGSGSYGWYQQKSPGSAPVTVIYYNYKRPSDIPS  RFSGSASGSTATLTITGVQAEDEAVYYCGSYDSTYAGMFGAGTTLTVL | AR | 0.97 |
| RFAR26 | GSGDSSGTEAA | LTQPSSVSANPGGTVEITCSGSSGSYGWYQQKSPGSAPVTLIYANTNRPSNIPS  RFSGSTSGSTATLTITGVQADDEAVYYCGSGDSSGTEAAFGAGTTLTVL | AR | 0.97 |
| RFAR27 | GSEDSSGAGYVGI | LTQPSSVSANPGETVKITCSGGSYGYSWHQQKSPGSAPVTVIYSSNQRPSDIPS  RFSGSTSGSTATLTITGVQADDEAVYFCGSEDSSGAGYVGIFGAGTTLTVL | AR | 0.96 |
| RFAR28 | GGFDSTDSGYAGI | LTQPSSVSANPGETVKITCSGSTSTYYGWYQQKSPGSAPVTLIYNNNNRPSDIPS  RFSGSTSGSTNTLTITGVRAEDEAVYYCGGFDSTDSGYAGIFGAGTTLTVL | AR | 0.96 |
| RFAR29 | GSADTKYVGI | LTQPSSVSANPGETVEITCSGDSSYYGWYQQKSPGSAPVTVIYDNTNRPSDIPS  RFSGSLSGSTNTLTITGVQVEDEAIYFCGSADTKYVGIFGAGTTLTVL | AR | 0.96 |
| RFAR30 | GSRDSSYLDSGI | LTQPSSVSANLGGTVKITCSGGGSYYGWYQQKAPGSAPVTLIYWNDNRPSDIPS  RFSGSKSGSTATLTITGVQADDEAVYYCGSRDSSYLDSGIFGAGTTLTVL | AR | 0.95 |

(continued)

| **Clone ID** | **Selected LCDR3** | **Mapped V_L_** | **Prediction** | **Probability** |
| --- | --- | --- | --- | --- |
| RFAR31 | GTWDSNTYA | LTQPSSVSANLGETVKITCSGGSGNYGWFQQKAPGSAPVTVIYYDDERPSNIPS  RFSGSTSGSTSTLTITGVQVEDEAVYFCGTWDSNTYAGIFGAGTTLTVL | AR | 0.95 |
| RFAR32 | GSYEDSSYVGI | LTQPSSVSANLGGTVKITCSGGSGSYGWFQQKSPGSVPVTVIYDSSSRPSDIPS  RFSGSKSGSTGTLTITGVQAEDEAVYFCGSYEDSSYVGIFGAGTTLTVL | AR | 0.95 |
| RFAR33 | GSYVSGKYDGI | LTQPSSVSANPGETAKITCSGGYRSYGWYQQKSPGSAPVTLIYSNNQRPSSIPS  RFSGSVSVFTHTLTITGVQAEDEAVYYCGSYVSGKYDGIFGAGTTLTVL | AR | 0.95 |
| RFAR34 | GTADSSTEAI | LTQPSSVSANPGETVKITCSGGSGRYGWFQQKSPGSAPVTVIYWDDERPSNIPS  RFSGSTSGSTNTLTITGVQVEDEAVYFCGTADSSTEAIFGAGTTLTVL | AR | 0.95 |
| RFAR35 | GSYDNTYAGI | LTQPSSVSANLGGTVEITCSGGSGSYGWYQQKAPGSAPVTVIYANTNRPSNIPS  RFSGSKSGSTNTLTITGVQAEDEAVYFCGSYDNTYAGIFGAGTTLTVL | AR | 0.95 |
| RFAR36 | GGYDSSSSSAV | LTQPSSVSANLGGTVKITCSGSSSNNYGWYQQKSPGSTPLTLIYWNDKRPSDIPS  RFSGSTSGSTATLTITGVQAEDEAVYFCGGYDSSSSSAVFGAGTTLTVL | AR | 0.95 |
| RFAR37 | GSYEDSNY | LTQPSSVSANPGETVEITCSGSRTGYGWFQQKSPGSAPVTLIYGSNKRPSNIPS  RFSGSKSGSTSTLTITGVQAEDEAVYFCGSYEDSNYFGAGTTLTVL | AR | 0.95 |
| RFAR38 | GSFDSSYSGI | LTQPSSVSANLGGTVKITCSGGSSGYYGWYQQKSPGSAPVTLIYSNNQRPSNIPS  RFSGSGSGSTGTLTITGVRAEDEAVYFCGSFDSSYSGIFGAGTTLTVL | AR | 0.95 |
| RFAR39 | GDWDSNI | LTQPSSVSANPGETVEITCSGDSNYYGWYQQKAPGSAPVTLIYANTNRPSNIPS  RFSGSGSGSTNTLTITGVQAEDEAVYYCGDWDSNIFGAGTTLTVL | AR | 0.95 |
| RFAR40 | GGYDSSSGA | LTQPSSVSANPGETVKITCSGGGSSRYYGWYQQKAPGSAPVTLIYDNTNRPSNIPS  RFSGSKSGSTATLTITGVQAEDEAVYFCGGYDSSSGAFGAGTTLTVL | AR | 0.94 |
| RFNR1 | GGYDGSTDAGI | LTQPSSVSANPGETVKITCSGSSSSYYGWYQQKSPGSAPVTLIYDNTNRPSDIPS  RFSGSKSGSTATLTITGVQADDEAVYFCGGYDGSTDAGIFGAGTTLTVL | NR | 0.00 |
| RFNR2 | GSTDSSYTDSL | LTQPSSVSANPGETVKITCSGGGSYDYGWYQQKSPGSAPVTVIYNNNKRPSDIPS  RFSGALSGSTATLTITGVQADDEAVYFCGSTDSSYTDSLFGAGTTLTVL | NR | 0.00 |
| RFNR3 | GNEDSSYAGI | LTQPSSVSANLGGTVEITCSGGSGSYGWFQQKAPGSAPVTLIYANTNRPSDIPS  RFSGSKSGSTATLIITGVQAEDEAVYFCGNEDSSYAGIFGAGTTLTVL | NR | 0.00 |
| RFNR4 | GNYADSSST | LTQPSSVSANPGETVKITCSGGTYNYGWYQQKSPGSAPVTVIYDNNKRPSDIPS  RFSGALSGSTATLTITGVQADDEAVYFCGNYADSSSTFGAGTTLTVL | NR | 0.00 |
| RFNR5 | GSADSSSAGI | LTQPSSVSANLGGTVKITCSGSSDSYGWYQQKSPGSAPVTLIYESNKRPSDIPS  RFSGSKSGSTGTLTITGVQAEDEAVYYCGSADSSSAGIFGAGTTLTVL | NR | 0.00 |
| RFNR6 | GSADSSGSGI | LTQPSSVSANPGETVKITCSGGGSYGWYQQKSPSSAPVTLIYTNTNRPSNIPS  RFSGSKSGSTGTLTITGVQAEDEAVYFCGSADSSGSGIFGAGTTLTVL | NR | 0.00 |
| RFNR7 | GSRDSSNVGI | LTQPSSVSANLGGTVEITCSGGGSYGWYQQKSPGSAPVTVIYWNDKRPSDIPS  RFSGSKSGSTGTLTITGVQAEDEAVYFCGSRDSSNVGIFGAGTTLTVL | NR | 0.00 |
| RFNR8 | GSYEGSSGIV | LTQPSSVSANPGETVKITCSGSSGSYGWYQQKSPGSAPVTVIYSNDKRPSDIPS  RFSGSASGSTATLTITGVQADDEAVYYCGSYEGSSGIVFGAGTTLTVL | NR | 0.00 |
| RFNR9 | GSRDSTDSLYVGI | LTQPSSVSANPGETVKITCSGGSSYYAWYQQKSPGSAPVTVIYYNDKRPSDIPS  RFSGSTSGSTSTLTITGVQADDEAVYFCGSRDSTDSLYVGIFGAGTTLTVL | NR | 0.00 |
| RFNR10 | GSADSSTDSGI | LTQPSSVSANPGGTVEITCSGGSSNYGWFQQKAPGSAPVTVIYNNNKRPSDIPS  RFSGSKSGSTGTLTITGVQADDEAVYFCGSADSSTDSGIFGAGTTLTVL | NR | 0.00 |
